# Prehabilitation programs for cancer patients: a systematic review of randomized controlled trials (protocol)

**DOI:** 10.1186/s13643-020-1282-3

**Published:** 2020-02-13

**Authors:** Jose F. Meneses-Echávez, Andrés F. Loaiza-Betancur, Víctor Díaz-López, Andrés M. Echavarría-Rodríguez

**Affiliations:** 1 Division for Health Services, Norwegian Institute of Public Health, Sandakerveien 24C, Building D11, 4th Floor, Office 434, Oslo, Norway; 2 Facultad de Cultura Física, Deporte y Recreación, Universidad Santo Tomás, Bogotá, Colombia; 3 University of Antioquia, Medellín, Colombia

**Keywords:** Cancer, Prehabilitation, Systematic review, Meta-analysis, Exercise

## Abstract

**Background:**

Around twenty million new cases and ten million of deaths were attributed to cancer in 2018. Physical exercise, as main component of prehabilitation programs, has been associated with clinical improvements in aerobic capacity, muscular strength, gait speed, and fewer postoperative complications. This systematic review aims to determine the benefits and harms of prehabilitation programs, mainly composed of physical exercise, compared with standard care for cancer patients.

**Methods/design:**

A librarian will systematically search for randomized controlled trials in the following databases: Cochrane Central Register of Controlled Trials (CENTRAL), MEDLINE (PubMed), and EMBASE. Two independent reviewers will independently screen the retrieved references, appraise the methodological quality of the included studies, and extract data. If possible, we will pool the data. We will evaluate the completeness of reporting of prehabilitation programs by using the CERT checklist, and the GRADE approach will be used to evaluate the quality of the evidence.

**Discussion:**

This systematic review will determine the benefits and harms of prehabilitation programs for cancer patients. We will provide a complete appraisal of the quality of the evidence, our confidence in the results, and completeness of reporting of the exercise interventions evaluated in the prehabilitation programs. Findings from this review will assist health care providers, patients, decision-makers, and international organizations to make informed decisions in this field.

**Systematic review registration:**

PROSPERO CRD42019125658

## Background

The National Cancer Institute in the USA defines cancer as a chronic disease in which abnormal cells divide without control, can invade nearby tissues, and can spread to other parts of the body through the blood and lymph systems [1]. GLOBOCAN reported 18.1 million new cases of cancer and 9.6 million of deaths in 2018 [2].

Cancer treatment might comprise surgery, chemotherapy, radiation therapy, immunotherapy, hematopoietic stem cell transplant, and hormone therapy; it all depends on the type of cancer and its stage [1]. In most cases, cancer treatments require surgery and postoperative care that lead to long periods of physical inactivity and deconditioning with loss of muscle function and a higher rate of medical complications [3]. Further, inactivity-induced loss of muscle mass predominantly affects the lower body musculature, being larger during the first days of inactivity [4–6]. Exercise interventions during and after medical treatment are associated with improvements in the quality of life [7] and decreases in fatigue and depression [7], and this is accompanied by lower tumor activity [8, 9] in cancer patients.

Cancer prehabilitation represents “a process on the continuum care that occurs between the time of cancer diagnosis and the beginning of acute treatment. It includes physical and psychological assessments that establish a baseline functional level, identifies impairments, and provides targeted interventions that improve a patient’s health to reduce the incidence and the severity of current and future impairments” [10]. This systematic review focuses on cancer prehabilitation programs that include exercise as the main component before surgical treatment.

Recent data from a Swedish cohort study showed that higher values of walking distance, leg strength, grip strength, gait speed, and inspiratory muscle strength are associated with fewer postoperative complications and shorter length of stay after abdominal cancer resection [11]. Prehabilitation programs might also improve lean mass and muscular strength, and delay the incidence of sarcopenia [12]. However, most research in cancer patients has focused on the impact of exercise interventions during the postoperative period (rehabilitation) [4, 13]. The period known as rehabilitation might be too late for people over 60 years with cancer, who are considered as a high-risk population because the physical capacity in this population is often diminished due to inactivity especially before surgery. High values of muscular strength and cardiorespiratory fitness in cancer patients may make them better prepared for recovery after surgery [13].

A recent systematic review conducted by Hamaker and colleagues [14] found relatively small benefits of prehabilitation programs and therefore questioned the investments that prehabilitation interventions require from both health care providers and patients. Unlike previous systematic reviews [14, 15], and in line with implications that emerged from their analyses, we will appraise the completeness of reporting of the prehabilitation programs in order to facilitate transferability of the findings, as well as grade the quality of the evidence.

## Review objectives

This systematic review aims to determine the benefits and harms of prehabilitation programs compared with standard care for cancer patients.

## Methods/design

This systematic review will be conducted according to the Cochrane Handbook [16] and reported in accordance with the guidelines of the Preferred Reporting Items for Systematic Reviews and Meta-Analyses (PRISMA) declaration [17]. This protocol has been written according to the PRISMA-P statement (Additional file 1) and is registered in the International Prospective Register of Systematic Reviews (PROSPERO registration number: CRD42019125658).

## Eligibility criteria

### Types of studies

We will include trials described as randomized (i.e., parallel, cluster, or crossover designs), even if methods used to generate the random sequence were unclear or unreported, or if the method of allocating participants was likely to be quasi-random (i.e., by alternation, date of birth, or similar pseudo-randomized method).

### Participants

Individuals older than 13 years old, survivors of any type of cancer, defined according to the Centers for Disease Control and Prevention (CDC), as anyone who has been diagnosed with cancer, from the time of diagnosis through the rest of life [18]. No restrictions will be made with regard to nationality, ethnicity, gender, duration of illness, or treatment setting.

### Interventions

We will consider cancer prehabilitation programs including exercise as the major component. The definition of prehabilitation programs is presented in the “Background” section. Exercise is understood as “any body movement causing an increase in energy expenditure that involves a planned or structured movement of the body performed in a systematic manner in terms of frequency, intensity, and duration and is designed to maintain or enhance health-related outcomes” [19]. Exercise interventions within the prehabilitation programs can involve different training modes, such as aerobic, resistance, and flexibility training, as well as yoga, Qi-gong, and Tai-Chi. We accept for inclusion different environments, such as aquatic- or land-based training [20]. Finally, we will not restrict on the type of exercise, dose, or materials used.

### Comparators

We will include comparator interventions defined as standard care or also named sham intervention, usual care, or wait-list control. We define standard care as the care a person would normally receive had they not been included in the research trial; this can include interventions such as medication, hospitalization, community nursing input, and/or day hospital.

### Outcomes

In order to provide a more comprehensive and clinically relevant set of outcome measures, the review team conducted a scoping search of recent systematic reviews in this field and mapped out the outcome measures explored among them. Three reviewers (AL, VD, and AE) conducted this process in October 2018. All team members reviewed and discussed the final set of outcomes to be included in this systematic review [3, 21–25]. Additional file 2 contains the results from this mapping exercise as well as the definitions of the prioritized outcomes.

#### Primary outcomes

- Health-related quality of life (HQoL)
- Muscular strength
- Postoperative complications

#### Secondary outcomes

- Average length of stay (ALOS)
- Handgrip strength
- Physical activity levels

### Search strategy

We will conduct a systematic search according to Chapter 6 of the Cochrane Handbook for Systematic Reviews of Interventions [16]. A research librarian will search Cochrane Central Register of Controlled Trials (CENTRAL), MEDLINE, and EMBASE. No restrictions will be applied for publication date or language. The search strategy used in MEDLINE is available online (Additional file 3). Two review authors will independently inspect the reference lists from key journals, identified articles, meta-analyses, and reviews of all types of exercise interventions for cancer patients, and will scrutinize all promising or potential references. Besides, one reviewer (JM) searches the following registers for ongoing studies:

- WHO International Clinical Trials Registry Platform (www.who.int/ictrp/)
- ClinicalTrials (https://clinicaltrials.gov/)

### Study selection

The retrieved references will be exported to Rayyan [26]. Pairs of reviewers will independently screen the references by using a pre-defined screening form. Disagreements will be solved by discussion or by involving a third reviewer.

### Data management and extraction

Pairs of reviewers will independently extract data from the studies (i.e., characteristics of each study, participants, interventions and comparators, outcomes, and study design). We will resolve disagreements by reaching consensus or by involving a third reviewer.

One reviewer (AL) will transfer data into Review Manager (RevMan) [27]. If necessary, we will attempt to contact authors through an open-ended request in order to obtain missing information or for clarification. We will note in the “Characteristics of included studies” table if outcome data are not reported in a usable way, when data are obtained directly from study authors, and times when data are transformed or estimated from a graph. In case both unadjusted and adjusted values are reported for the same outcome, we will extract the adjusted values. If data are analyzed on an intention-to-treat (ITT) sample and another sample (e.g., per-protocol, as-treated), we will extract ITT data.

### Risk of bias assessment

Two reviewers (AL and VD) will independently assess risk of bias by using criteria described in the Cochrane Handbook for Systematic Reviews of Interventions [16]. This set of criteria is based on evidence of associations between potential overestimation of effect and the level of risk of bias of the trial that may be due to aspects of sequence generation, allocation concealment, blinding, incomplete outcome data, selective reporting, and other sources of bias. For other sources of bias, we will consider potential sources of bias such as baseline inequities despite randomization. We will rate each criterion as low, high, or unclear risk of bias. We will select the criterion “unclear risk” when the review authors’ ability to determine the potential for bias could not be determined by information on the primary article or contact with author. In such cases, we will revise the assessments if the authors responded to our requests for more information.

### Data synthesis

We will calculate risk ratio (RR) and its 95% confidence interval (CI) for binary outcomes, whereas continuous data will be expressed as group post-test means and standard deviations (SDs) to calculate effect sizes. We will communicate effect sizes preferentially in the form of mean differences (MDs) and 95% CIs, but when different scales were used to measure the same outcome, we will calculate standardized mean differences (SMDs) instead, with corresponding 95% CIs.

In order to perform a meta-analysis, we will perform arithmetic conversions of the point estimates of outcomes: (a) to express results in the same units (e.g., centimeters will be transformed to millimeters) or (b) to resolve differences in the direction of the scale (when scores derived from scales with higher score indicating greater health were combined with scores derived from scales with high scores indicating greater disease). These conversions will enable calculation of relative change, pooling of data, or both. When back-translation of SMD effect sizes is not possible, we will use Cohen’s guidelines (no effect < 0.2, small effect = 0.2 to 0.49, moderate effect = 0.5 to 0.79, large effect ≥ 0.80) [28] to report the magnitude of the effect and help with the interpretation of SMDs.

We understand that there is no closed argument for preference for use of fixed-effect or random-effects models to conduct meta-analysis. The fixed-effect model assumes that the intervention effects are identical across the studies, which is unlikely in most scenarios. On the contrary, the random-effects model incorporates an assumption that the different studies are estimating different, yet related, intervention effects [16]. This often seems to be true to us, and the random-effects model takes into account differences between studies, even if there is no statistically significant heterogeneity (*I*^2^ < 50%) [16]. Therefore, we will preferably choose a random-effects model for all analyses, and we will further study heterogeneity through sensitivity analysis.

### Planned subgroup analyses

#### Type of cancer or clinical state

We plan to report data on subgroups to explore the relative effects (as represented by the MD or SMD) for participants in the same age group, and clinical state or type of cancer, such as breast, prostate, or colorectal. This is mainly due to the expected large number of trials in each category. We may also conduct subgroup analysis for different characteristics of the intervention (i.e., length in weeks and/or setting).

#### Mixed interventions

We will explore the individual effects of mixed prehabilitation programs, which combine exercise with other parallel interventions, such as diet, psychological approaches, or any pharmacological option.

#### Investigation of heterogeneity

We will report if heterogeneity across studies is high. First, we will investigate whether data have been entered correctly. Secondly, if data are correct, we will inspect the forest plots visually and remove outlying studies successively to see if homogeneity is restored. When unanticipated clinical or methodological heterogeneity is obvious, we will simply state hypotheses regarding these for future reviews or versions of this review. We do not anticipate undertaking analyses relating to these.

#### Sensitivity analysis (risk of bias assessment)

We will analyze the effects of excluding trials that are at high risk of bias across one or more of the criteria (see the “Risk of bias assessment” section) for the meta-analysis of the primary outcomes.

#### Quality of the evidence: GRADE approach

We will follow the GRADE Working Group grades of evidence to prepare “Summary of findings” tables for the six major outcomes [29]. We will integrate analysis of quality of evidence and the magnitude of effect of the interventions. The GRADE approach considers the risk of bias and the body of evidence to rate the quality of the evidence into one of four levels:

*High certainty*: We are very confident that the true effect lies close to that of the estimate of the effect.

*Moderate certainty*: We are moderately confident in the effect estimate—the true effect is likely to be close to the estimate of the effect, but there is a possibility that it is substantially different.

*Low certainty*: Our confidence in the effect estimate is limited—the true effect may be substantially different from the estimate of the effect.

*Very low certainty*: We have very little confidence in the effect estimate—the true effect is likely to be substantially different from the estimate of effect.

#### Reporting of exercise interventions in the prehabilitation programs

We will use the CERT tool (Consensus on Exercise Reporting Template) to evaluate the completeness of reporting of exercise interventions [30, 31]. As stated by authors, “The CERT has the potential to increase clinical uptake of effective exercise programmes, enable research replication, reduce research waste and improve patient outcomes” [30, 31]. Two independent reviewers (AE and VD) will apply the tool to the included trials.

## Discussion

This systematic review will establish the benefits and harms of prehabilitation programs compared with standard care for cancer patients. To our knowledge, and in terms of methodological rigor, this review represents the most complete evidence synthesis in this field. A research librarian with broad experience in evidence synthesis will undertake systematic literature searches, and outcome selection process is informed by a scoping search. Furthermore, and unlike other reviews in this field, the research methods for the present systematic review will cover an assessment of the quality of the evidence alongside a detailed appraisal of the completeness of reporting of exercise interventions. Information derived from these steps might serve as a facilitator for evidence-informed decision-making processes and assist health care providers when implementing findings from this systematic review [32]. Thus, our findings will contribute upon the strengthening of exercise as a crucial component of multidisciplinary cancer care.

## Supplementary information

**Additional file 1:** PRISMA-P 2015 Checklist.

**Additional file 2:** Outcome measures prioritization: scoping methodological exercise.

**Additional file 3:** PubMed/MEDLINE search strategy.

## Data Availability

Not applicable.

## Abbreviations

CERT: Consensus on Exercise Reporting Template
GRADE: Grading of Recommendations, Assessments, Development and Evaluation
MD: Mean difference
PRISMA-P: Preferred Reporting Items for Systematic Review and Meta-Analyses Protocol
PROSPERO: Prospective Register of Systematic Reviews
SMD: Standardized mean difference
