# Supplementary material for: Prehabilitation programs for cancer patients: a systematic review of randomized controlled trials (protocol)
Source: Syst Rev. 2020 Feb 13;9:34. doi: 10.1186/s13643-020-1282-3 (PMC7020348; doi:10.1186/s13643-020-1282-3)
Supplement: Supplementary file 2 — Additional file 2: Outcome measures prioritization: scoping methodological exercise. [file 13643_2020_1282_MOESM2_ESM.docx]

**Supplementary file 2**

**Outcome measures prioritization: scoping methodological exercise**

Two reviewers searched MEDLINE during October 2018 in order to identify systematic reviews related to our research question. All reviewers checked the included reviews and extracted information on the outcome measures (e.g., frequency of reporting and measurement tools). The outcome measures are prioritized according to their inclusion across the analyzed systematic reviews. We provide definitions for each outcome and the accepted measurement tools.

| Outcome measure | N° systematic reviews | Definition and measurement tools |
| --- | --- | --- |
| Health-Related Quality Of Life (HQOL) | 4^(1-4)^ | World Health Organization (WHO) defines quality of life as an individual's perception of their position in life in the context of the culture and value systems in which they live and in relation to their goals, expectations, standards and concerns. It is a broad ranging concept affected in a complex way by the person's physical health, psychological state, personal beliefs, social relationships and their relationship to salient features of their environment. HRQoL can be measured with different tools, such as EORTC-QLQ 30FACT-G SF-36, WHOQOL-100 and the WHOQOL-BREF (5-8). |
| Muscle strength | 3^(1,4,9)^ | Understood as a health-related component of physical fitness, which is related to the amount of external force exerted by a muscle. Muscle strength can be measured with different tools, such as one-repetition maximum, Borg Scale, modified Borg Scale, OMNI-Resistance Scale (10-13). |
| Average Length of Stay (ALOS): | 3^(1,3,4)^ | This outcome refers to the average number of days that patients spend in hospital. It is generally measured by dividing the total number of days stayed by all inpatients during a year by the number of admissions or discharges (14,15). |
| Physical activity levels | 2^(1,16)^ | This outcome refers to the amount of physical activity practiced by an individual, which can be reported in days, weeks, or months (17,18). The following tools are considering for the analysis: (NILSSON H), LASA Physical Activity Questionnaire, Active Tracer (AC-301) Acelerometer (17-19) |
| Handgrip strength | 2^(3,16)^ | Reflects the maximum strength derived from combined contraction of either extrinsic or intrinsic hand muscles, which lead to the flexion of hand joints (20). Low handgrip strength levels (defined as grip strength <26 kg for men and <16 kg for women) is considered an important risk factor for morbidity after gastric cancer surgery (21), and is associated with all cause and cardiovascular disease mortality than do systolic blood pressure or total physical activity (22). |
| Postoperative complications | 2^(4,9)^ | These are defined as any deviation from the normal postoperative course. Postoperative complications can be measured with different tools, such as The Clavien-Dindo classification of surgical complications (15,23) |

**References**

1. Singh F, Newton RU, Galvão DA, Spry N, Baker MK. A systematic review of pre-surgical exercise intervention studies with cancer patients. Surg Oncol. 2013;22(2):92–104.

2. Treanor C, Kyaw T, Donnelly M. An international review and meta-analysis of prehabilitation compared to usual care for cancer patients. J Cancer Surviv. 2018;12(1):64–73.

3. Bruns ERJ, van den Heuvel B, Buskens CJ, van Duijvendijk P, Festen S, Wassenaar EB, et al. The effects of physical prehabilitation in elderly patients undergoing colorectal surgery: a systematic review. Color Dis. 2016;18(8):O267–77.

4. Piraux E, Caty G, Reychler G. Effects of preoperative combined aerobic and resistance exercise training in cancer patients undergoing tumour resection surgery: A systematic review of randomised trials. Surg Oncol. 2018;27(3):584–94.

5. Arraras Urdaniz Juan Ignacio, Villafranca Iturre Elena, Arias de la Vega Fernando, Domínguez Domínguez Miguel Angel, Lainez Milagro Nuria, Manterola Burgaleta Ana et al. The EORTC quality of life questionnaire QLO-C30 (version 3.0): Validation study for spanish prostate cancer patients. Arch. Esp. Urol. 61( 8 ): 949-954.

6. Weitzner MA, Meyers CA, Gelke CK, Byrne KS, Levin VA, Cella DF. The functional assessment of cancer therapy (FACT) scale. Development of a brain subscale and revalidation of the general version (FACT‐G) in patients with primary brain tumors. Cancer. 1995;75(5):1151–61.

7. Pinar R. Reliability and construct validity of the SF-36 in Turkish cancer patients. Quality of Life Research. 2005;14(1):259-64.

8. O’Carroll RE, Smith K, Couston M, Cossar JA, Hayes PC. A comparison of the WHOQOL-100 and the WHOQOL-BREF in detecting change in quality of life following liver transplantation. Qual Life Res. 2000 Feb;9(1):121–4.

9. van Rooijen SJ, Engelen MA, Scheede-Bergdahl C, Carli F, Roumen RMH, Slooter GD, et al. Systematic review of exercise training in colorectal cancer patients during treatment. Scand J Med Sci Sport. 2018;28(2):360–70.

10. Caspersen CJ, Powell KE, Christenson GM. Physical activity, exercise, and physical fitness: definitions and distinctions for health-related research. Public Health Rep 100(2):126-131. 1985;100(2).

11. Chen MJ, Fan X, Moe ST. Criterion-related validity of the Borg ratings of perceived exertion scale in healthy individuals: A meta-analysis. J Sports Sci. 2002;20(11):873–99.

12. Robertson RJ, Goss FL, Rutkowski J, Lenz B, Dixon C, Timmer J, et al. Concurrent validation of the OMNI perceived exertion scale for resistance exercise. Med Sci Sports Exerc. 2003;35(2):333–41.

13. Dong-il Seo, Eonho Kim, Fahs CA, Rossow L, Young K, Ferguson SL, et al. Reliability of the one-repetition maximum test based on muscle group and gender. Journal of Sports Science & Medicine. 11(2):221–5.

14. OECD. Organisation for Economic Cooperation and Development. Health at a glance. 2011.

15. Dindo D, Demartines N, Clavien PA. Classification of surgical complications: A new proposal with evaluation in a cohort of 6336 patients and results of a survey. Ann Surg. 2004;240(2):205–13.

16. Yang A, Sokolof J, Gulati A. The effect of preoperative exercise on upper extremity recovery following breast cancer surgery: A systematic review. Int J Rehabil Res. 2018;41(3):189–96.

17. Kushi LH, Doyle C, McCullough M, Rock CL, Demark-Wahnefried W, Bandera E V, et al. American Cancer Society Guidelines on nutrition and physical activity for cancer prevention: reducing the risk of cancer with healthy food choices and physical activity. CA Cancer J Clin. 2008;62(1):30–67.

18. Rock CL, Doyle C, Demark-Wahnefried W, Meyerhardt J, Courneya KS, Schwartz AL et al. Nutrition and physical activity guidelines for cancer survivors. CA Cancer Journal for Clinicians. 2012 Jan 1;62(4):242-274. <https://doi.org/10.3322/caac.21142>.

19. Gillis C, Fenton TR, Sajobi TT, Minnella EM, Awasthi R, Loiselle S-È, et al. Trimodal prehabilitation for colorectal surgery attenuates post-surgical losses in lean body mass: A pooled analysis of randomized controlled trials. Clinical nutrition. 2019;38(3):1053-60.

20. Norman K, Stobäus N, Gonzalez MC, Schulzke JD, Pirlich M. Hand grip strength: Outcome predictor and marker of nutritional status. Clin Nutr. 2011;30(2):135–42.

21. Sato T, Aoyama T, Hayashi T, Segami K, Kawabe T, Fujikawa H, et al. Impact of preoperative hand grip strength on morbidity following gastric cancer surgery. Gastric Cancer. 2016;19(3):1008–15.

22. Celis-Morales CA, Welsh P, Lyall DM, Steell L, Petermann F, Anderson J, et al. Associations of grip strength with cardiovascular, respiratory, and cancer outcomes and all cause mortality: prospective cohort study of half a million UK Biobank participants. Bmj. 2018;361:k1651.

23. Clavien PA, Barkun J, de Oliveira ML, Vauthey JN, Dindo D, Schulick RD, et al. The Clavien-Dindo classification of surgical complications: five-year experience. Ann Surg. 2009;250(2):187–96.
