# Supplementary material for: Prehabilitation programs for cancer patients: a systematic review of randomized controlled trials (protocol)
Source: Syst Rev. 2020 Feb 13;9:34. doi: 10.1186/s13643-020-1282-3 (PMC7020348; doi:10.1186/s13643-020-1282-3)
Supplement: Supplementary file 3 — Additional file 3: PubMed/MEDLINE search strategy. [file 13643_2020_1282_MOESM3_ESM.docx]

**Supplementary file 3**

PubMed/MEDLINE search strategy

((neoplasms$ [Text Word] OR lymphoma$ [Text Word] OR radiotherapy[Text Word] OR Bone Marrow Transplantation[Text Word] OR cancer$[Text Word] OR leukaemia[Text Word] OR leukemia[Text Word] OR (tumour$ OR tumor$)[Text Word] OR malignan$[Text Word] OR neutropeni$[Text Word] OR carcino$[Text Word] OR adenocarcinoma$[Text Word] AND (prehabilitation[Title/Abstract] OR preoperative[Title/Abstract] OR pre-operative[Title/Abstract] OR presurg*[Title/Abstract] OR pre-surg*[Title/Abstract] OR before surg*[Title/Abstract] OR before operat*[Title/Abstract]) AND (exercise[MeSH Terms] OR exercis*[Title/Abstract] OR rehabilitation[MeSH Terms] OR rehabilitat*[Title/Abstract] OR aerobic*[Title/Abstract] OR endurance[Title/Abstract] OR treadmill[Title/Abstract] OR walking[MeSH Terms] OR walk*[Title/Abstract] OR breathing exercises[MeSH Terms] OR respiratory muscle training[Text Word] OR bicycl*[Title/Abstract] OR cycling*[Title/Abstract] OR physiotherap*[Title/Abstract] OR physical therap*[Title/Abstract]))
